# Monitoring of Colorectal Cancer Screening Adherence Among African American Patients of North Florida Community Health Centers

**DOI:** 10.1007/s13187-025-02738-4

**Published:** 2025-10-24

**Authors:** John S. Luque, Gebre-Egziabher Kiros, Askal A. Ali, Sabrina L. Dickey, Matthew Vargas, Deloria R. Jackson, Ryan Mohorne, Tanvee Doddi, Kristin Wallace, Clement K. Gwede

**Affiliations:** 1 College of Pharmacy and Pharmaceutical Sciences, Institute of Public Health, Florida A&M University, 1515 South Martin Luther King Jr. Blvd., Tallahassee, FL 32307 USA; 2 College of Nursing, Florida State University, 98 Varsity Way, Tallahassee, FL 32306 USA; 3 Department of Public Health Sciences, College of Medicine, Medical University of South Carolina, 68 President Street, Charleston, SC 29425 USA; 4 Hollings Cancer Center, Medical University of South Carolina, 86 Jonathan Lucas Street, Charleston, SC 29425 USA; 5 Division of Population Sciences, Department of Health Outcomes and Behavior, Moffitt Cancer Center, 12902 USF Magnolia Dr., Tampa, FL 33612 USA

**Keywords:** Colorectal cancer, Cancer screening, African Americans, Survey research

## Abstract

In the United States, colorectal cancer (CRC) mortality rates are higher in African Americans compared to non-Hispanic whites, partly due to advanced stage cancer diagnosis. Timely CRC screening helps to increase CRC early detection and survival in this population. The objective of this monitoring study was to survey African American patients of Community Health Centers (CHC) in north Florida and to monitor CRC screening adherence (either stool-based or colonoscopy) after they had completed a clinical trial testing a screening education intervention. Seventy-nine African American patients who were between the ages of 45 and 64 years old at the time of initial trial recruitment completed a 24-month follow-up survey, and 44% reported stool-based CRC screening in the last year. Results from the general estimating equations (GEE) model found there was a statistically significant difference in CRC screening adherence by study arm at 24 months where the intervention group was less likely to be up to date than the usual care control (OR = 0.60, 95% CI 0.43–0.83). Married or partnered participants (OR = 1.52, 95% CI 1.31–1.77) and employed participants (OR = 1.34, 95% CI 1.17–1.53) were more likely to be adherent to screening, but female participants were less likely to be adherent (OR = 0.76, 95% CI 0.70–0.82). Participants with higher mistrust in doctors were less likely to have completed screening (OR = 0.93, 95% CI 0.88–0.99). The findings suggest the importance of screening outreach to unmarried, unemployed, female African Americans. The results of this monitoring study indicate one-on-one cancer education with a community health advisor and education on the availability and functionality of electronic patient portals have potential for increasing adherence to recommended CRC screening. The study has implications for measuring CRC screening adherence in community settings.

## Introduction

Colorectal cancer (CRC) is the second leading cause of mortality in the US for men and women combined [1]. CRC health disparities persist between African Americans and non-Hispanic Whites (NHW) [2]. African Americans have higher CRC incidence, present with more advanced stage cancers, and experience lower survival rates than NHW; moreover, they have the second highest CRC incidence in the US when comparing all major racial/ethnic groups [3]. Compared to NHW men and women, African American men have 20% higher incidence rates, and African American women have 14% higher incidence rates [3]. What drives incidence rates includes behavioral risk factors such as obesity, an unhealthy diet, and low physical activity levels [4]. When comparing CRC mortality rates for African Americans and NHW, there are greater cancer health disparities than with incidence rates. This statistic is reflected in higher CRC survival rates for NHW (65%) compared to African Americans (59%) [3]. Reasons for higher CRC mortality in African Americans are attributed to differences in access and quality of care, number of comorbidities, environmental factors, social determinants of health, and tumor characteristics [3, 5–7].

There are different CRC screening modalities, but the only screening that removes polyps during the screening procedure is the colonoscopy. Screening with colonoscopy decreases the risk of cancer metastasis through the removal of adenomatous polyps. One modeling study indicated that CRC mortality rates would be reduced by 19% if African Americans received timely follow-up colonoscopy following abnormal stool-based test results and had adenoma detection rates similar to NHW [2].

The US Preventive Services Task Force recommends colorectal cancer screening for average-risk adults between the ages of 45 and 75 [8]. For adults aged 50 years and older, 70% of African Americans are up-to-date with CRC screening compared to 71% of NHW, which is more similar than it was in the past, when African Americans had lower screening adherence rates [3]. While national screening rates have been increasing over time, screening adherence is substantially lower among medically underserved, uninsured, lower education, and ethnically minoritized populations [9]. A national study reported that differences in screening rates by education level were much more pronounced for colonoscopy or sigmoidoscopy compared to non-invasive CRC screening [9]. Non-invasive CRC screening such as stool-based tests is more accessible for patients than screening colonoscopy, since patients can complete the test at home and either use the mail or deliver the test back to the healthcare facility for laboratory processing.

In another national study, there was an analysis of cancer screening use in federally qualified health centers (FQHC) and the authors reported CRC screening adherence rates ranging from 25% in Alabama to 61% in Maine [10]. The study estimated that hypothetically optimizing screening to 100% could increase CRC screening among ethnically minoritized populations by 8.5% [10]. In Florida, the setting of the current study, FQHCs contributed 182,594 people (11.7%) to the total number of the underscreened general population (1,554,666) eligible for CRC screening [10]. Therefore, approximately 40% of the FQHC patient population in Florida who were eligible for CRC screening received screening [10]. In community health centers (CHC) in the US, stool-based tests are the default test used for their screening programs; moreover, a national study reported 44% of CHC patients were up-to-date with CRC screening [11]. Common barriers for CHCs to implement high-quality screening programs include lack of laboratory testing capacity, low patient knowledge of CRC screening options, and low capacity for leveraging patient navigation, electronic reminders, or electronic medical records to notify patients of testing intervals [11].

This study is a monitoring study and a follow-up study to the “Test Up Now Education Program” (TUNE-UP) randomized behavioral clinical trial [12] (Clinical trial registration: NCT04304001). The aim of the TUNE-UP trial was to test a brief community health advisor (CHA) intervention adapted from the Centers for Disease Control and Prevention “Screen to Save” intervention to boost stool-based CRC screening in an underscreened, African American, CHC patient population. Patients from the trial were recontacted and recruited to the monitoring study to assess repeat annual stool-based CRC screening in this CHC patient cohort at 24-month and 36-month post initial trial recruitment. The monitoring study continued to assess the research participants in the cohort using the same questions and study measures as the baseline survey study [13].

## Methods

The objective of this study was to test predictors at the 24-month follow-up assessment of being up to date with annual stool-based CRC screening using survey questions on participant demographics, as well as study variables such as CHC site and original trial study arm. This article reports on the 24-month survey outcomes with this patient cohort and compares findings with 12-month survey outcomes, which were collected at the end of the behavioral clinical trial (Table 1). The study was approved by the Florida A&M University Institutional Review Board (IRB#2,010,561–6).

**Table 1:** Descriptive statistics of sociodemographic characteristics, cancer perceptions, and colorectal cancer screening adherence of African Americans in longitudinal study at 12- and 24-month follow-up

Characteristics	12 months (*n* = 93)^a^		24 months (*n* = 79)^a^	
	Percent distribution	Percent adherence	Percent distribution	Percent adherence
Original trial study arm				
Intervention	48.4	64.4	48.1	42.1
Control	51.6	64.6	51.9	48.8
Age group (years)				
45–54	50.0	55.6	36.7	41.4
55–67	50.0	73.3	63.3	49.0
Gender				
Male	45.2	71.4	43.0	50.0
Female	54.8	58.8	57.0	42.2
Marital status				
Single/separated/widowed/divorced	75.3	65.7	75.9	41.7
Married/living with a partner	24.7	60.9	24.1	57.9
Education				
< 12 years	13.3	50.0	12.7	40.0
12 years or high school	25.6	65.2	24.1	47.4
Some college, technical, or higher	61.1	65.5	63.2	46.0
Employment status				
Employed	36.6	58.8	37.9	46.7
Not employed	63.4	67.8	62.1	44.9
Community Health Center				
Community Health Center 1	60.2	64.3	59.5	51.1
Community Health Center 2	39.8	64.9	40.5	37.5
Health insurance coverage				
Yes	65.6	67.2	81.0	26.7
No	34.4	59.4	19.0	50.0
Have a regular provider				
Yes	67.7	60.3	83.5	47.0
No	32.3	73.3	16.5	38.5
Self-reported health status				
Excellent/very good	23.3	66.7	20.8	43.8
Good	38.9	54.3	40.3	41.9
Fair/poor	37.8	73.5	38.9	53.3
Family history of cancer				
Yes	65.2	66.7	58.2	41.3
No	34.8	61.1	41.8	45.5
Chances of getting cancer				
Very unlikely/unlikely	30.1	60.7	39.2	45.2
Neither unlikely nor likely	39.8	81.1	37.9	46.7
Very likely/likely	30.1	46.4	22.9	44.4
Worry about cancer				
Not at all/slightly	41.9	66.7	32.9	38.5
Somewhat	28.0	61.5	32.9	38.5
Moderately/extremely	30.1	64.3	34.2	59.3
I would rather not know my chance of getting cancer				
Strongly agree/somewhat agree	24.7	56.5	29.1	47.8
Strongly disagree/somewhat disagree	75.3	67.4	70.9	44.6
If you were diagnosed with cancer, obtaining follow-up care would be difficult				
Yes	19.4	72.2	13.9	36.4
No	80.6	62.7	86.1	47.1
A doctor has discussed different CRC screening tests with you				
Yes	66.7	69.4	74.7	54.2
No	33.3	54.8	25.3	25.0

^a^Total may not sum to number of 93 or 79 for some questions because of missing or “not applicable response” data

### Data Collection and Study Measures

Survey questions were adapted from the Health Information Trends National Survey (HINTS) on health insurance coverage, having a regular provider, history of cigarette smoking, self-reported health status, chronic health conditions, family history of cancer, beliefs and attitudes about cancer and colorectal cancer, and adherence to colorectal cancer screening [14]. There were also questions from validated scales and measures on healthcare communication, mistrust in doctors, CRC perceived susceptibility, CRC worry, negative cancer beliefs, CRC screening self-efficacy, CRC knowledge, and the multi-construct African American cultural survey (MAACS) [15–19]. The dependent variable for the analysis was a binary variable for whether the participant was adherent to CRC screening (either stool-based screening within the last year or colonoscopy screening within the last 10 years).

### Participant Recruitment

In order to be eligible for the original trial, study participants needed to satisfy the following criteria: (1) 45–64 years of age, (2) African American, (3) Florida resident, (4) not up-to-date with CRC screening (> 9 months since stool test, > 9 years since last colonoscopy, or > 4 years since last flexible sigmoidoscopy), and (6) currently a patient of one of two different local CHCs in north Florida. Since we already had the participants’ contact information, we were able to contact the trial participants and provide informed consent for their participation in the monitoring study over the phone. Participant recruitment began in May 2023 and lasted until March 2025 for the 24-month follow-up period. The project coordinator administered the surveys in person to study participants, completed the gift card receipt form, and provided a $30 retail store gift card for each participant’s time in the study. Participants also received a tailored educational brochure, which featured photos of African Americans and relevant messages about the importance of CRC screening [20].

### Data Management

The project graduate research assistant entered the paper surveys in the REDCap research management software platform, and data entry validity was checked by the project coordinator for each individual survey in the software. REDCap is a secure web application and is hosted on a local server at the university [21]. The data were exported to a statistical package for further analysis.

### Statistical Analysis

SAS 9.4 (Cary, NC) was used for data analysis. Data quality was assessed via data validation and consistency check procedures. Descriptive statistics and frequency distributions were produced for all variables. Next, bivariate analyses were conducted for key variables and scales to compare any changes from the 12-month follow-up surveys to the 24-month follow-up surveys using paired *t*-tests. In addition, the same key variables and scales were used to compare differences between participants who were and were not up to date with CRC screening (either stool-based screening within the last year or colonoscopy screening within the last 10 years). Multilevel logistic regression analysis was performed using a generalized estimating equations (GEE) model to compare the original intervention group with the original control group participants and adjusting for other covariates and confounding variables. The GEE model is a statistical method for modeling clustered data. Since study participants were randomized within CHCs in this study, there is a clustering of data. The GEE model was used to analyze the clustered data and considered the clustering of participants (level-1) within a Community Health Center (level-2) in estimating the regression parameters. The model was fitted using the GENMOD procedure in SAS with the REPEATED statement and exchangeable working correlation structure. Our final model included the following variables: study arm, age, sex, marital status, education, employment, trust in doctors, and Community Health Center. Significance tests were two-sided, and the significance level was set to *α* = 0.05.

## Results

At the 24-month follow-up period, a total of 79 African American patients who were between the ages of 45 and 64 years old from CHCs in north Florida at the time of initial trial recruitment completed the follow-up survey. At 24-month follow-up, 75% of the sample reported ever completing a stool-based CRC screening at home and having discussed CRC screening test with a doctor; however, only 44% of participants had completed CRC screening in the last year. A lower percentage of participants (32%) had received a colonoscopy. For CRC screening adherence, at 12-month follow-up there were 60 participants up to date with either stool-based screening or colonoscopy (53 stool-based only, seven colonoscopy only), or 65% of the 93 study participants at the first 12-month follow-up period. At 24-month follow-up, there were 36 participants up to date with either stool-based screening or colonoscopy (32 stool-based only, four colonoscopy only), or 46% of the 79 study participants.

Categorizing participants from the original randomized controlled trial, 48% of participants were randomized to the intervention group and 52% were randomized to the control group. The average age of participants was 56.2 (SD = 5.1). There were more female participants (57%) compared to male participants (43%), and a higher percentage of males (50%) were adherent to screening than females (42%). There were fewer participants reporting being married or living with a partner (24%) compared to other relationship categories, and married or partnered participants had a higher percentage of CRC screening adherence (58%). There were more participants in the higher education category (63%) compared to the other categories, and fewer participants were currently employed (38%). There were more participants who were patients of Community Health Center 1 (60%) compared to Community Health Center 2 (40%), and participants in Community Health Center 1 had a higher percentage of patients adherent to CRC screening at the 24-month follow-up (51%) than Community Health Center 2 (38%). There was a greater percentage of participants who had health insurance (81%) compared to those without (19%), and more participants reported they had a regular provider (84%). Moreover, 14% of participants believed obtaining follow-up care would be difficult if they were diagnosed with cancer. Over half (58%) of participants reported a family history of cancer, and one-third of participants (34%) were either “moderately” or “extremely” worried about a potential cancer diagnosis. There was a higher percentage of participants who were adherent to CRC screening (54%) for the 75% of participants who answered “yes” to the question about whether a doctor had discussed different CRC screening tests with them compared to those who had not had this conversation with their doctor.

There were 75 survey participants who responded to both the 12-month and 24-month surveys. Bivariate analysis of scale measures was conducted to test if there were any changes in mean scores between the two time periods for the overall sample. There was a statistically significant difference for the scale of negative cancer beliefs (*t*(74) = 2.82, *p* = 0.006), indicating more negative cancer beliefs represented by lower mean scores at the 24-month follow-up compared to the 12-month follow-up. There was no statistically significant difference for the other scales, which included: healthcare communication, mistrust in doctors, CRC perceived susceptibility, CRC worry, CRC screening self-efficacy, CRC knowledge, and MAACS subscales (Table 2).

**Table 2:** Responses for cancer-related beliefs and attitudes of African Americans in longitudinal study at 12-months and 24-months follow-up

Characteristics	12 months (*M* ± *SD*) (*n* = 93)	24 months (*M* ± *SD*) (*n* = 79)	*p* values
*Healthcare communication scale*^*a*^	**11.26 ± 5.09**	**10.97 ± 5.32**	00.633
How often did they give you the chance to ask all the health-related questions you had?			
How often did they give you the attention you needed to your feelings and emotions?			
How often did they involve you in decisions about your health care as much as you wanted?			
How often did they make sure you understood the things you needed to do to take care of your health?			
How often did they explain things in a way you could understand?			
How often did they spend enough time with you?			
How often did they help you deal with feelings of uncertainty about your health or health care?			
*Mistrust in doctors scale*^*b*^	**22.11 ± 8.18**	**21.32 ± 7.82**	0.308
Your doctor does whatever it takes to get you the care you need			
Sometimes your doctor cares more about convenience than your health care			
Your doctor’s medical skills are not as good as they should be			
Your doctor is extremely thorough and careful			
You completely trust your doctor’s decisions about what’s best for you			
Your doctor is totally honest about all treatment options available to you			
Your doctor only thinks about what is best for you			
Sometimes your doctor doesn’t pay full attention to what you’re telling them			
You have no worries about putting your life in your doctor’s hands			
You completely trust your doctor			
*CRC perceived susceptibility*^*c*^	**8.36 ± 2.45**	**8.20 ± 2.11**	0.534
I believe the chance I might develop CRC is high			
I think it is very likely I will develop CRC or polyps			
I believe the chance I will develop colorectal polyps is high			
*CRC worry*^*c*^	**5.80 ± 1.54**	**5.92 ± 1.57**	0.547
I am afraid of having an abnormal screening result			
I am worried that screening will show that I have CRC or polyps			
*Negative cancer beliefs*^*c*^	**11.01 ± 2.71**	**10.28 ± 2.20**	0.006*
It seems like everything causes cancer			
There’s not much you can do to lower your chances of getting cancer			
It’s so hard to know which cancer prevention recommendations to follow			
In adults, cancer is more common than heart disease			
*CRC screening self-efficacy*^*c*^	**7.56 ± 2.33**	**7.85 ± 2.18**	0.273
Arranging my schedule for CRC screening is easy			
Finding time for CRC screening would be difficult			
Going through CRC screening would be difficult			
Going through CRC screening would be easy			
*CRC knowledge*^*d*^	**10.17 ± 1.70**	**10.53 ± 2.11**	0.115
*MAACS measures and subscales*^*e*^			
* MAACS religiosity (6 items)*	**10.72 ± 3.54**	**11.09 ± 3.62**	0.308
* MAACS mistrust/discrimination (9 items)*	**29.09 ± 5.97**	**28.17 ± 4.70**	0.125
* MAACS privacy (6 items)*	**22.40 ± 4.18**	**22.09 ± 3.64**	0.461
* MAACS ethnic Identity (7 items)*	**18.35 ± 5.01**	**18.57 ± 3.57**	0.617
* MAACS collectivism (5 items)*	**12.93 ± 4.51**	**12.24 ± 3.45**	0.099
* MAACS empowerment (6 items)*	**12.48 ± 4.19**	**12.57 ± 3.74**	0.845
* MAACS male role (4 items)*	**9.59 ± 3.68**	**8.93 ± 3.48**	0.095

Values in bold represent summary scale scores and *n* = 75 for comparison of two time periods

*M* mean, *SD* standard deviation, *MAACS* multi-construct African American cultural survey

^a^Only participants who had a regular provider answered these questions. Response categories for these items are the following: 1 =* always*; 2 = *usually*; 3 = *sometimes*; 4 = *never*

^b^Response categories for these items are the following: 1 =* strongly agree*; 2 = *agree*; 3 = *neutral*; 4 = *disagree*; 5 = *strongly disagree*

^c^Response categories for these items are the following: 1 = *strongly agree*; 2 = *agree*; 3 = *disagree*; 4 = *strongly disagree*

^d^Responses for the knowledge questions were recorded for correct and incorrect responses, with a minimum score of 0 and a maximum score of 14

^e^Response categories for these items are the following: 1 = *strongly disagree*; 2 = *disagree*; 3 = *neutral*; 4 = *agree*; 5 = *strongly agree*

*p < 0.05

For the 12-month data on CRC adherence to screening guidelines—either stool-based testing within the last year or colonoscopy within the last 10 years—in the intervention arm there were 29 participants who were CRC screening adherent (64.4%) out of 45 participants, and in the control arm there were 31 participants who were CRC screening adherent (64.6%) out of 48 participants. For the 24-month data on CRC screening adherence, in the intervention arm, there were 16 participants who were CRC screening adherent (42.1%) out of 38 participants, and for the control arm, there were 20 participants who were CRC screening adherent (48.8%) out of 41 participants. The chi-square test for CRC screening adherence from 12 to 24 months was statistically significant (*Χ*^2^ = 4.43, *p* = 0.035), showing that almost half of the patients (48%) who were adherent at 12 months were still adherent at 24 months, and 17% of patients who were non-adherent at 12 months remained non-adherent at 24 months. However, 27% of patients who were adherent at 12 months were no longer adherent at 24 months, suggesting a drop in adherence rates (*data not shown*). There was also a statistically significant difference for the healthcare communication scale (*t*(67) = 3.25, *p* = 0.002), indicating higher mean scores on the scale of health communication with their doctor for those up to date with CRC screening compared to those not up to date with screening (*data not shown*). When comparing participants up to date with CRC screening compared to those not up to date with CRC screening, there were no statistically significant differences between mean values for the following scales: mistrust in doctors, CRC perceived susceptibility, CRC worry, CRC screening self-efficacy, CRC knowledge, and the MAACS subscales.

Results from the GEE model found there was a statistically significant difference in adherence to CRC screening by study arm at 24 months (OR = 0.60, 95% CI 0.43–0.83) favoring the control group, but screening adherence was similar at 12 months. In terms of demographic characteristics, married or partnered participants (OR = 1.52, 95% CI 1.31–1.77) and employed participants (OR = 1.34, 95% CI 1.17–1.53) were more likely to be adherent to CRC screening, but female participants were less likely to be adherent (OR = 0.76, 95% CI 0.70–0.82). Participants with higher scores on the doctors’ mistrust scale were less likely to have stool-based CRC screening tests at home at the 24-month follow-up (OR = 0.93, 95% CI: 0.88–0.99). Finally, CHC site was a factor, with patients from Community Health Center 1 being more likely to be adherent to CRC screening at the 24-month follow-up (OR = 2.60, 95% CI 1.83–3.72) (Table 3).

**Table 3:** Results from a GEE model estimating the likelihood of adherence to CRC screening after 12 and 24 months of the intervention

	12 months (*n* = 93)		24 months (*n* = 79)	
Variables	OR	95% CI	OR	95% CI
Study arm				
Control	REF		REF	
Intervention	1.21	0.81–1.79	0.60^**^	0.43–0.83
Age group				
55–67 yrs	REF		REF	
45–54 yrs	0.47^**^	0.27–0.83	0.85	0.18–4.04
Gender				
Male	REF		REF	
Female	0.48	0.15–1.48	0.76^**^	0.70–0.82
Marital status				
Single/separated/widowed/divorced	REF		REF	
Married/living with a partner	0.99	0.50–1.99	1.52^**^	1.31–1.77
Education				
< 12 years or high school	REF		REF	
Some college, technical, or higher	1.37	0.64–2.95	1.27	0.37–4.36
Employment status				
Not employed	REF		REF	
Employed	0.49	0.17–1.45	1.34^**^	1.17–1.53
Mistrust in doctors	1.03^*^	1.01–1.06	0.93^*^	0.88–0.99
Community Health Center				
Community Health Center 2	REF		REF	
Community Health Center 1	0.91^**^	0.85–0.97	2.60^**^	1.83–3.72
Quasi-likelihood statistic	113.84^**^		106.55^**^	

*CI* confidence interval, *OR* odds ratio, *REF* reference category

*p < 0.05, **p < 0.01

## Discussion

The study findings for this two-year cohort of African American patients attending CHCs identified marital status, employment status, gender, mistrust in doctors, and CHC patient home as predictors of CRC screening adherence. At the 24-month follow-up, participants who were married or partnered had a 16 percentage point higher adherence to CRC screening compared to other relationship status groups. This result is similar to our baseline survey findings, which also indicated married or partnered individuals were more likely to have ever received CRC screening [13]. According to a national survey, 69% of married participants completed CRC screening compared to 53% of never married individuals, which mirrors our findings [22]. Other studies using national survey data have also found an association between marital status and adherence to CRC screening recommendations [23, 24]. In addition, our survey identified employed participants as more likely to be adherent to CRC screening compared to unemployed participants. Other studies in the US have demonstrated that people who are employed, and would be more likely to have employee benefits such as health insurance and paid sick leave, would be more likely to complete timely CRC screening [25, 26].

Both at 12-month and 24-month follow-up, participants in the younger age category had a lower percentage of participants who were adherent to CRC screening, and at 12-month follow-up, younger age was a predictor of lower CRC screening adherence. This finding has implications for public health practice in terms of timely screening messaging to individuals 45 to 49 years of age. A qualitative study with African American populations in Kentucky reported low awareness of stool-based CRC screening and the importance of culturally relevant outreach from trusted messengers [27]. Another qualitative study with African American populations in Wisconsin also identified low awareness of stool-based CRC screening options among younger patients [28]. There is growing recognition of beginning CRC screening at age 45, especially for those with a family history of cancer; moreover, the latest CRC statistics indicate the increasing number of early-onset CRC in the US population, including in African Americans [29].

In terms of overall adherence, participants were recruited to the original trial only if they responded that they were not up to date with CRC screening. At the 12-month follow-up, 65% of participants were up to date with CRC screening; however, there was a drop-off at the 24-month follow-up and only 44% of participants were adherent to CRC screening, which is similar to the average for CHC patients nationwide [11]. There was a 7-point percentage point difference in CRC screening adherence favoring the original control group from the previous trial. This finding might be explained by the fact that there was no educational boost by a community health advisor for the intervention group, since this study was a follow-up monitoring study and did not use the same clinical trial design as the initial study. Any residual effect from the brief educational intervention delivered to intervention arm participants did not affect the primary outcome—CRC screening adherence—2 years after receiving the educational intervention in the trial. However, all participants in this monitoring study did receive the tailored educational brochure about the importance of regular CRC screening, yet this educational boost occurred at the same time as the 24-month follow-up survey. Our study surveys the participants again at the 36-month follow-up, so we will monitor if screening rates continue to decrease without an additional educational intervention other than the brochure. Based on the study results, we can conclude that the CHC text patient appointment reminder system should be more closely monitored or other strategies considered to ensure patients adhere to recommended CRC screening. The results of our initial trial demonstrated a boost in screening for the intervention group following the 3-month follow-up after receiving one-on-one cancer education with a community health advisor to educate the patient about the importance of scheduling and completing stool-based CRC screening [12]. A systematic review examining CRC screening interventions for African Americans in both community and clinical studies suggested patient navigation and tailored educational materials increased stool-based CRC screening [30]. Therefore, we recommend that CHCs implement the community health advisor intervention with the tailored brochure as part of their usual care to increase their CRC screening adherence rates.

There were also possible clinic-specific factors that affected screening, since Community Health Center 1 patients were significantly more up to date with screening at 24 months compared to Community Health Center 2 patients, even though screening levels were 51% and 36% for the health centers, respectively. The main findings demonstrate that while there was an initial boost in screening for both health centers after 12 months, with 65% of patients being up to date, adherence levels dropped by 20% after another year of follow-up. These results point to the challenges of annual stool-based screening programs, which have been identified in other studies [31, 32]. CHCs may consider including the CRC screening reminder with the annual physical exam reminder.

CRC screening initiatives led by CHCs must build and maintain trust between providers and patients. The 10-item mistrust in doctors scale emerged as a significant predictor of CRC adherence in the multilevel logistic regression model. One systematic review identified medical mistrust as a predictor of lower CRC screening rates in African Americans [33]. In our study, at 24-month follow-up, lower mistrust in doctors was a predictor of greater CRC screening adherence among the African American patients in the cohort.

In our study, 75% of participants had discussed CRC screening with their doctor, and 54% of those who responded “yes” to this question were adherent to CRC screening compared to the 25% of participants who had not discussed CRC screening with their doctor and were not adherent to CRC screening. A large survey study with African Americans in Los Angeles reported that participants who received a CRC screening recommendation from their provider were significantly more likely to obtain screening [34]. This finding from our study was a positive outcome that more patients were having discussions with their providers about the importance of CRC screening. Further, it was evident that some participants were either: (1) not informed of their CRC screening test result; (2) not aware that their screening result could be viewed through their online patient portal; or (3) may not have the technological skills to access their patient portal. There is also the opportunity to provide educational resources in the patient portal that might be tailored by the provider to the patient’s specific health needs. Therefore, there is an opportunity for patient education around the use of electronic patient portals and accessing one’s test results.

## Limitations

Survey participant attrition was the most significant challenge encountered in the study. From a participant response rate of approximately 81% for the 12-month follow-up survey in the randomized clinical trial, we then experienced a 69% survey completion rate with the monitoring study’s 24-month follow-up survey. Decreased survey participation was due to loss of contact with participants (disconnected phone, not reachable by mail), non-response to contact attempts, ineligible due to non-Florida resident status, declined participation, and, in two cases, mortality. Another limitation was self-report of screening. In the survey, participants were only asked about stool-based screening adherence and colonoscopy adherence; however, some patients may have received the stool DNA test and may not have known the difference between this test and the annual fecal immunochemical test (FIT) in terms of screening frequency. Future studies with CHCs need to adjust screening questions to account for the longer recommended screening interval of the stool DNA test of 2 to 3 years to better measure CRC screening adherence. Despite study attrition, the study was able to recruit and retain a sizable proportion of participants from the original trial, as well as recruit a number of these participants to a focus group study to discuss their experiences with CRC screening, which will be discussed elsewhere.

## Conclusion

The study findings suggest the challenges of CRC screening adherence among African American patients of CHCs. Certain factors, including age, marital status, gender, employment status, and mistrust in doctors, were predictors of CRC screening adherence in the study population. Quarterly partner meetings between CHC representatives and project investigators indicate the important role that CHCs play in the implementation of CRC stool-based screening programs to improve screening in their communities. CHCs provide stool-based screening to their patients; however, referrals and finding funding for uninsured patients to receive timely colonoscopy screening remain a challenge for these patients.

## Data Availability

The datasets collected and analyzed during the current study are available from the corresponding author on reasonable request with a data sharing agreement.

## References

[1] Siegel RL, Kratzer TB, Giaquinto AN, Sung H, Jemal A (2025) Cancer statistics, 2025. CA Cancer J Clin 75(1):10–45. 10.3322/caac.21871 39817679 PMC11745215

[2] Alagoz O, May FP, Doubeni CA, Fendrick AM, Vahdat V, Estes C et al (2024) Impact of racial disparities in follow-up and quality of colonoscopy on colorectal cancer outcomes. J Natl Cancer Inst 116(11):1807–1816. 10.1093/jnci/djae140 39044335 PMC11542987

[3] Saka AH, Giaquinto AN, McCullough LE, Tossas KY, Star J, Jemal A, Siegel RL (2025) Cancer statistics for African American and Black people, 2025. CA Cancer J Clin 75(2):111–140. 10.3322/caac.21874 39976243 PMC11929131

[4] Siegel RL, Wagle NS, Cercek A, Smith RA, Jemal A (2023) Colorectal cancer statistics, 2023. CA Cancer J Clin 73(3):233–254. 10.3322/caac.21772 36856579

[5] Carethers JM, Doubeni CA (2020) Causes of socioeconomic disparities in colorectal cancer and intervention framework and strategies. Gastroenterology 158(2):354–367. 10.1053/j.gastro.2019.10.029 31682851 PMC6957741

[6] Roy S, Davis SN, Luque JS, Gwede CK (2023) Colorectal cancer. In: Coughlin SS, Williams LB, Akintobi TH (eds) Black Health in the South. Johns Hopkins University Press, Baltimore, MD, pp 307–331

[7] Carethers JM (2024) The Jeremiah Metzger lecture: environmental influences on colorectal cancer. Trans Am Clin Climatol Assoc 134:181–199 39135583 PMC11316861

[8] U. S. Preventive Services Task Force, Davidson KW, Barry MJ, Mangione CM, Cabana M, Caughey AB et al (2021) Screening for colorectal cancer: US Preventive Services Task Force recommendation statement. JAMA 325(19):1965–77. 10.1001/jama.2021.6238 34003218

[9] Mondragon Marquez LI, Dominguez Bueso DL, Gonzalez Ruiz LM, Liu JJ (2023) Associations between sociodemographic factors and breast, cervical, and colorectal cancer screening in the United States. Cancer Causes Control : CCC 34(12):1073–1084. 10.1007/s10552-023-01758-z 37486400

[10] Amboree TL, Montealegre JR, Parker SL, Garg A, Damgacioglu H, Schmeler KM et al (2024) National breast, cervical, and colorectal cancer screening use in federally qualified health centers. JAMA Intern Med 184(6):671–679. 10.1001/jamainternmed.2024.0693 38683574 PMC11059050

[11] Huguet N, Hodes T, Holderness H, Bailey SR, DeVoe JE, Marino M (2022) Community health centers’ performance in cancer screening and prevention. Am J Prev Med 62(2):e97–e106. 10.1016/j.amepre.2021.07.007 34663549 PMC8748316

[12] Luque JS, Kiros GE, Vargas MA, Ali A, Tawk R, Jackson DR et al (2025) Effectiveness of a community health advisor colorectal cancer screening educational intervention on stool test completion in an African American primary care patient population: a pragmatic randomized controlled trial. BMC Glob Public Health 3(1):47. 10.1186/s44263-025-00168-4 40450376 PMC12126889

[13] Luque JS, Kiros GE, Vargas M, Jackson DR, Matthew OO, Austin TD et al (2023) Association of preventive care attitudes and beliefs with colorectal cancer screening history among African American patients of community health centers. J Cancer Educ 38(6):1816–1824. 10.1007/s13187-023-02337-1 37442915 PMC10787027

[14] National Cancer Institute, Health information national trends survey (2018) http://hints.cancer.gov/instrument.aspx. Accessed 19 June 2018

[15] Vernon SW, Myers RE, Tilley BC (1997) Development and validation of an instrument to measure factors related to colorectal cancer screening adherence. Cancer Epidemiol Biomarkers Prevent: Publ Am Assoc Cancer Res Cosponsored Am Soc Preventive Oncol 6(10):825–832 9332766

[16] Vernon SW, Meissner H, Klabunde C, Rimer BK, Ahnen DJ, Bastani R et al (2004) Measures for ascertaining use of colorectal cancer screening in behavioral, health services, and epidemiologic research. Cancer epidemiology, biomarkers & prevention : a publication of the American Association for Cancer Research, cosponsored by the American Society of Preventive Oncology 13(6):898–905 15184243

[17] Thompson VL, Bugbee A, Meriac JP, Harris JK (2013) The utility of cancer-related cultural constructs to understand colorectal cancer screening among African Americans. J Public Health Res 2(2):e11. 10.4081/jphr.2013.e11 25170482 PMC4147735

[18] Morris NS, MacLean CD, Chew LD, Littenberg B (2006) The single item literacy screener: evaluation of a brief instrument to identify limited reading ability. BMC Fam Pract 7:21. 10.1186/1471-2296-7-21 16563164 PMC1435902

[19] Balkrishnan R, Dugan E, Camacho FT, Hall MA (2003) Trust and satisfaction with physicians, insurers, and the medical profession. Med Care 41(9):1058–1064. 10.1097/01.MLR.0000083743.15238.9F 12972845

[20] Vargas MA, Matthew OO, Jackson DR, Austin T, Tawk R, Wallace K, et al (2021) Adaptation of a community health advisor intervention to increase colorectal cancer screening among African Americans in the Southern United States. Cancer Health Disparities 5. https://companyofscientists.com/index.php/chd/article/view/204 PMC8893047 35252768

[21] Harris PA, Taylor R, Thielke R, Payne J, Gonzalez N, Conde JG (2009) Research electronic data capture (REDCap)–a metadata-driven methodology and workflow process for providing translational research informatics support. J Biomed Inform 42(2):377–381. 10.1016/j.jbi.2008.08.010 18929686 PMC2700030

[22] Hanske J, Meyer CP, Sammon JD, Choueiri TK, Menon M, Lipsitz SR et al (2016) The influence of marital status on the use of breast, cervical, and colorectal cancer screening. Prev Med 89:140–145. 10.1016/j.ypmed.2016.05.017 27215758

[23] Baeker Bispo J, Lee H, Jemal A, Islami F (2025) Associations of social support, living arrangements, and residential stability with cancer screening in the United States. Cancer Causes Control : CCC 36(2):157–169. 10.1007/s10552-024-01913-0 39422870

[24] Shah SK, Narcisse MR, Hallgren E, Felix HC, McElfish PA (2022) Assessment of colorectal cancer screening disparities in U.S. men and women using a demographically representative sample. Cancer Res Commun 2(6):561–569. 10.1158/2767-9764.crc-22-0079 36381661 PMC9645794

[25] Stimpson JP, Park S, Morenz AM, Gurley T, Wilson FA (2025) Examining employment status, paid sick leave, and access to care in relation to colorectal cancer screening among U.S. workers: a structural equation modeling approach. Cancer Control 32:10732748251347731. 10.1177/10732748251347731 40464597 PMC12138219

[26] Song EY, Swanson J, Patel A, MacDonald M, Aponte A, Ayoubi N et al (2021) Colorectal cancer risk factors and screening among the uninsured of Tampa Bay: a free clinic study. Prev Chronic Dis 18:E16. 10.5888/pcd18.200496 33630731 PMC7938966

[27] Kruse-Diehr AJ, Cegelka D, Holtsclaw E, Stapleton J, Burnett C, Wood R et al (2023) Barriers and facilitators to stool-based screening for colorectal cancer among Black Louisville residents. J Cancer Educ 38(3):1050–1058. 10.1007/s13187-022-02231-2 36301412 PMC10501315

[28] Keiser E, Corbett AM, Chido-Amajuoyi O, Antoine A, Stehman C, Dorn I et al (2025) Acceptability of stool-based DNA colorectal cancer screening among Black/African-American patients served by federally qualified health centers. Journal of cancer education : the official journal of the American Association for Cancer Education. 10.1007/s13187-025-02631-0 PMC12971806 40307656

[29] Brim H, Reddy CS, Chirumamilla L, Oskrochi G, Deverapalli M, Rashid R et al (2025) Trends and symptoms among increasing proportion of African Americans with early-onset colorectal cancer over a 60-year period. Dig Dis Sci 70(1):168–176. 10.1007/s10620-024-08739-5 39586927 PMC12129110

[30] Roy S, Dickey S, Wang HL, Washington A, Polo R, Gwede CK, Luque JS (2021) Systematic review of interventions to increase stool blood colorectal cancer screening in African Americans. J Community Health 46(1):232–244. 10.1007/s10900-020-00867-z 32583358 PMC7313439

[31] Murphy CC, Halm EA, Skinner CS, Balasubramanian BA, Singal AG (2020) Challenges and approaches to measuring repeat fecal immunochemical test for colorectal cancer screening. Cancer Epidemiol Biomarkers Prev 29(8):1557–1563. 10.1158/1055-9965.EPI-20-0230 32457184 PMC7416474

[32] Arevalo M, Sutton SK, Abdulla R, Christy SM, Meade CD, Roetzheim RG, Gwede CK (2024) Longitudinal adherence to annual colorectal cancer screening among Black persons living in the United States enrolled in a community-based randomized trial. Cancer 130(9):1684–1692. 10.1002/cncr.35169 38150285 PMC11009071

[33] Adams LB, Richmond J, Corbie-Smith G, Powell W (2017) Medical mistrust and colorectal cancer screening among African Americans. J Community Health 42(5):1044–1061. 10.1007/s10900-017-0339-2 28439739 PMC5654700

[34] Cobb S, Ekwegh T, Adinkrah E, Ameli H, Dillard A, Kibe LW, Bazargan M (2022) Examining colorectal cancer screening uptake and health provider recommendations among underserved middle aged and older African Americans. Health Promot Perspect 12(4):399–409. 10.34172/hpp.2022.52 36852204 PMC9958235

